# When fate follows age: unequal centrosomes in asymmetric cell division

**DOI:** 10.1098/rstb.2013.0466

**Published:** 2014-09-05

**Authors:** Jose Reina, Cayetano Gonzalez

**Affiliations:** 1 Institute for Research in Biomedicine (IRB-Barcelona), Baldiri Reixac 10, Barcelona 08028, Spain; 2 Institució Catalana de Recerca i Estudis Avançats (ICREA), Passeig Lluís Companys 23, Barcelona 08010, Spain

**Keywords:** centrosome, centriole, stem cell, asymmetric cell division

## Abstract

A strong correlation between centrosome age and fate has been reported in some stem cells and progenitors that divide asymmetrically. In some cases, such stereotyped centrosome behaviour is essential to endow stemness to only one of the two daughters, whereas in other cases causality is still uncertain. Here, we present the different cell types in which correlated centrosome age and fate has been documented, review current knowledge on the underlying molecular mechanisms and discuss possible functional implications of this process.

## 1. Introduction

Paraphrasing T. Dobzhansky archcited quote one could say: ‘nothing in cell biology makes sense except in the light of development’ [1]. Admittedly an overstatement—like the original—this sentence does hold a great deal of truth. In Metazoa, cells have a time, a place and a function, and all cellular processes are orchestrated to fulfil the needs dictated by these critical coordinates. Metabolic pathways, the cell cycle, gene expression or cell shape cannot escape this principle; and, indeed, neither can centrosomes.

Self-renewing asymmetric division (SRAD) is a type of asymmetric cell division (ACD) in which one daughter retains the identity of the mother cell and can therefore undergo SRAD repeatedly. Current knowledge of the molecular cell biology of ACD and SRAD, including the mechanisms of asymmetric cell fate, spindle assembly and the function of cell asymmetry in development and disease, has been abundantly covered in the literature [2–15] and will not be further introduced. This article focuses exclusively on the topic of centrosome asymmetry in cells that divide by SRAD, be they stem cells (SCs) or progenitors.

Because the founding articles appeared only 7 years ago [16–18] and because the total count of articles published until now is still below 10 (four of which have been published in the last 12 months), it is fair to state offhand that the subject of centrosome asymmetry in SRAD is in its infancy: only a few cell types have been observed and the molecular details are still sketchy [19,20]. Yet, the stereotyped behaviour that mother and daughter centrosomes display in these cells, so markedly different and tightly linked to the unequal fate of the resulting daughter cells has caught the attention of cell and developmental biologists alike.

## 2. Intrinsical centriolar asymmetry

**Figure 1.**
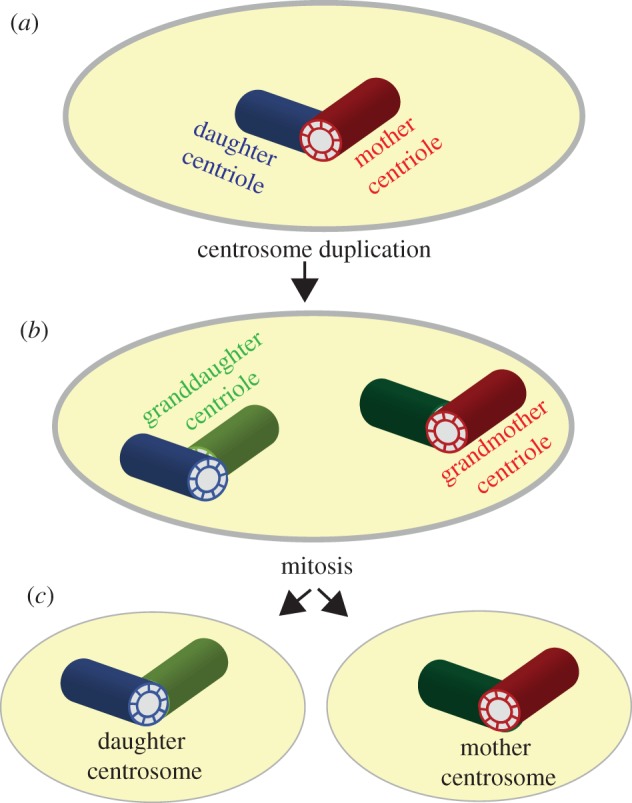
Centrosome asymmetry. (*a*) Diplosomes contain one mother centriole (red) and its daughter centriole (blue). (*b*) Following centrosome duplication, two new daughter centrioles are assembled (dark and pale green) and the cell contains a lineage of four centrioles. (*c*) The mother centrosome is earmarked by carrying the only grandmother centriole within this lineage of four centrioles. Centrosome segregation during mitosis results in daughter cells that are always asymmetric in terms of centriole age.

Asymmetry is built-in in the semiconservative nature of centriole duplication; in any given diplosome (the centriole pair of a centrosome), one centriole is mother to the other, hence older (figure 1*a*, red). Typically, diplosome duplication results in a lineage of four centrioles that includes one grandmother centriole (figure 1*b*, red) that identifies the mother centrosome (figure 1*c*). The other centrosome, which happens to contain the only granddaughter centriole of the lineage of four, is referred to as the daughter centrosome (figure 1*c*). Indeed, and most importantly, asymmetry goes beyond age because mother and daughter centrioles also present notable differences in molecular composition, ultrastructure and function [15,21–24]. For example, in mammals several proteins such as outer dense fibre protein 2, ninein and centrosomal protein 164 [21,25–28] are found only in the mother centriole while others like centrobin (CNTROB) are daughter centriole specific [21,29–32]. Moreover, in these cells, only the mother centriole contains ultrastructural appendages and can form cilia [21,25,26,33–35].

The obvious corollary derived from the above is that far from exception, ACD is the rule as far as centriole segregation is concerned; in any mitosis, only one daughter cell inherits the mother centrosome (figure 1). This peculiarity, although acknowledged by researchers in the field, has been traditionally disregarded in as much as mitosis was defined as the process where a single cell divides to produce two identical daughters. The remarkable finding of a link between centrosome asymmetry and the unequal fate of daughter cells, first reported in *Drosophila* male germline SCs (mGSCs) [16] and soon afterwards in larval neuroblasts (NBs) [36,37], put an end to this view.

## 3. Unequal centrosomes in unequal daughter cells

### (a) In *Drosophila*

**Figure 2.**
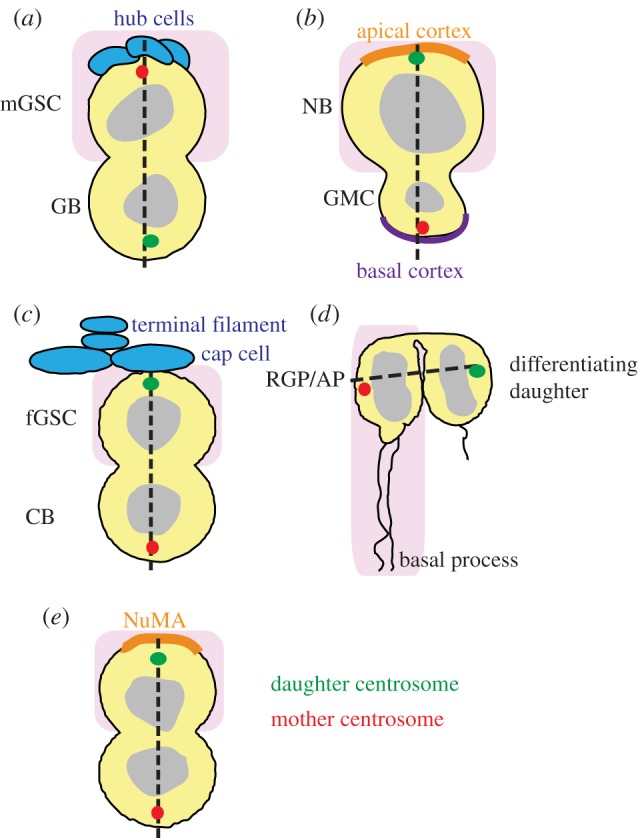
Centriole age and fate in self-renewing ACD. Position of mother (red) and daughter (green) centrosomes during SRAD in five cell types. (*a*) *Drosophila* mGSC; hub cells are labelled blue; differentiating gonialblast is labelled as GB. (*b*) *Drosophila* NB; the apical and basal sides of the cortex are labelled brown and purple, respectively; the differentiating ganglion mother cell is labelled as GMC. (*c*) *Drosophila* fGSC; terminal filament and cap cells are labelled blue; differentiating cytoblast is labelled as CB. (*d*) Mice and rat RGPs/APs. (*e*) Neuroblastoma cell line; the NuMA crescent is labelled brown. Pink areas mark the position of the cells that retain stemness and dotted lines represent the stemness/differentiation axes that, in all these five cell types, coincide with the position of centrosomes at mitosis.

*Drosophila* mGSCs are arranged around hub cells that serve as a niche that provides stemness signals (figure 2*a*). SRAD in mGSCs occurs along a proximo-distal axis defined by the hub (figure 2, dotted line): the renewed mGSC daughter cell always remains proximal to the hub, while the differentiating daughter is delivered distally [38]. In 2007, Yamashita and co-workers showed that the mother centrosome stays proximal-cortex bound throughout interphase, organizes the spindle pole that is nearest the hub and is retained by the renewed mGSC. Concomitantly, the daughter centrosome migrates distally where it organizes the second spindle pole and is delivered to the daughter cell that enters the spermatogenesis programme [16]. Yamashita's observations established for the first time that for some SCs, centrosome asymmetry is an integral part of SRAD and revealed a tantalizing link between centrosome age and fate. The stage was then set for the ‘immortal centrosome’ hypothesis proposing that upon SRAD, the daughter cell with more proliferative potential retains the mother centrosome [39], which may perhaps contribute to the asymmetric segregation of proteins, RNAs, organelles [40] or old-versus-new DNA strands (i.e. the ‘immortal strand hypothesis’) [41]. A similar observation had previously been made in the budding yeast *Saccharomyces cerevisiae* where the longer lived budding daughter inherits the old spindle pole body (the yeast functional counterpart of animal centrosomes) [42].

Two independent reports published later in the same year revealed features of the centrosome cycle in *Drosophila* larval NBs that were closely reminiscent of those described in mGSCs (figure 2*b*). *Drosophila* NBs undergo repeated rounds of SRAD, each of which generates a daughter cell fated to divide once more before terminal differentiation (ganglion mother cell; GMC) and a renewed NB. The two reports published in 2007 showed that one of the NB's centrosomes remains apical-cortex bound during interphase and is retained by the renewed NB, whereas the other migrates to the basal side of the NB and is delivered to the differentiating daughter [17,18]. Which of these were the mother and daughter centrosomes could not be established at that time. These studies also revealed aspects of the centrosome cycle that underscored the unequal nature of centrosomes in these cells. Live microscopy recordings obtained by following a centrosomin-green fluorescent protein (CNN-GFP) fusion as a pericentriolar material (PCM) reporter, and Asterless-(ASL)-GFP to trace centrioles, showed that centrosome asymmetry has a very early onset in NBs; only 10 min after cytokinesis, the centrosome splits in two centrosomes that remain markedly different through the entire cell cycle. The centrosome that stays at the apical cortex retains as much CNN as a mature mitotic centrosome, whereas the other loses most, if not all CNN immediately after splitting and moves across the cell for most of the cell cycle until settling down near the basal cortex, prior to mitosis. The CNN-rich centrosome organizes a very prominent microtubule network throughout interphase, which is remarkable since the interphase microtubule cytoskeleton is almost non-existent in most *Drosophila* diploid cell types, including indeed, both the neuroectodermal cells from which NBs derive, and GMCs, the NB daughters that do not retain stemness [30,43,44]. The same stereotyped centrosome behaviour was observed a few years later in embryo NBs [45], an exception made of the first cell cycle during which NBs delaminate [46].

Thus, despite notable differences in tissue architecture and stemness signalling, a clear theme common to mGSCs and NBs emerged. In a ‘differentiation axis’, defined as the straight line that bisects the two unequally fated daughters during cell division (figure 2, dotted line), one centrosome is permanently located at the stem-proximal end (i.e near the hub in mGSCs; near the apical cortex in NBs) and is inherited by the SC, whereas the other centrosome migrates distally and is delivered to the differentiating daughter cell. By extension, the observations from NBs were taken as further, albeit indirect, evidence in support of the immortal centrosome hypothesis, a conclusion that was to be proved wrong only a few years later.

In 2010, using the relative greater loading of GFP-pericentrin-AKAP450 centrosomal targeting (PACT) in the mother centriole as a reporter, Conduit & Raff [36] concluded that the apical-cortex-bound centrosome, which is the one retained by the NB, is the daughter, not the mother as the immortal centrosome hypothesis would have predicted. Januschke *et al*. [37] confirmed these findings by photo conversion experiments with which they were able to unequivocally trace mother centrosomes. This study also showed that in NBs, at the time of splitting, mother and daughter centrosomes contain only one centriole rather than a diplosome. Very recent results published by the Yamashita laboratory demonstrate that correlated centriole age and fate also operates in the female germline, and that like NBs, female GSCs (fGSCs) inherit the daughter centrosome [47] (figure 2*c*).

Altogether, these observations falsify the immortal centrosome hypothesis in *Drosophila* NBs and fGSCs, but provide further evidence to substantiate the hypothesis that centrosome maturation and fate are tightly correlated during SRAD. Moreover, these observations call for reassessing mother–daughter equivalence in *Drosophila*. As referred to before, in mammals, appendages and satellites are distinct attributes of mother centrioles that bear mother-centriole-specific functions such as PCM retention and cilia formation [25]. For decades, the lack of these features in *Drosophila* centrioles has been taken as suggestive of the absence of maturation-dependent centriolar functions in this species [48]. Age-dependent functional centrosome asymmetry in male and fGSCs and NBs shows that mother and daughter centrioles are unequal in *Drosophila*. Consistently, recent reports are starting to reveal some aspects of ultrastructural centriole dimorphism in flies [49,50].

### (b) In rodents

SRAD of radial glia progenitor cells (RGPs; also known as apical progenitors, APs; referred to as RGPs/APs henceforth) in the ventricular zone in mice produce self-renewed RGPs/APs and differentiating cells [51]. In 2009, Wang *et al*. [52] showed that RGPs/APs retain the mother centrosome (figure 2*d*). Furthermore, they assayed the effect of ninein depletion in these cells. Ninein is a coiled-coil centrosomal protein that localizes to the subdistal appendages that characterize mature centrioles and plays a key role in the anchorage of γTUB-containing complexes and microtubule minus-ends [26,53–55]. They found that ninein depletion disrupts the asymmetric inheritance of mother and daughter centrosomes and results in loss of RGPs/APs, suggesting a function for preferential inheritance of the mother centrosome in RGPs/APs maintenance in the developing mammalian neocortex [52]. The same effect was observed upon depletion of ninein in rat embryos [56]. Retention of the mother centrosome upon SRAD in RGPs/APs has also been documented by Paridaen *et al*. [57].

### (c) In human cancer cells

In 2012, Izumi & Kaneko [58] showed that in human neuroblastoma cells that assemble a NuMA cortical crescent at mitosis, the nuclear mitotic apparatus (NuMA) retaining daughter preferentially inherits the daughter centrosome (figure 2*e*). NuMA is the vertebrate homologue of *Drosophila* mushroom body defect, which during SRAD polarizes to the apical cortex that is inherited by the NB. However, in this case, the greater proliferation potential of the NuMA retaining cell is still hypothetical, suggested only by analogy to mouse dermal epidermis cells [59] and *Drosophila* NBs [60–62].

## 4. The mechanistic insight

**Figure 3.**
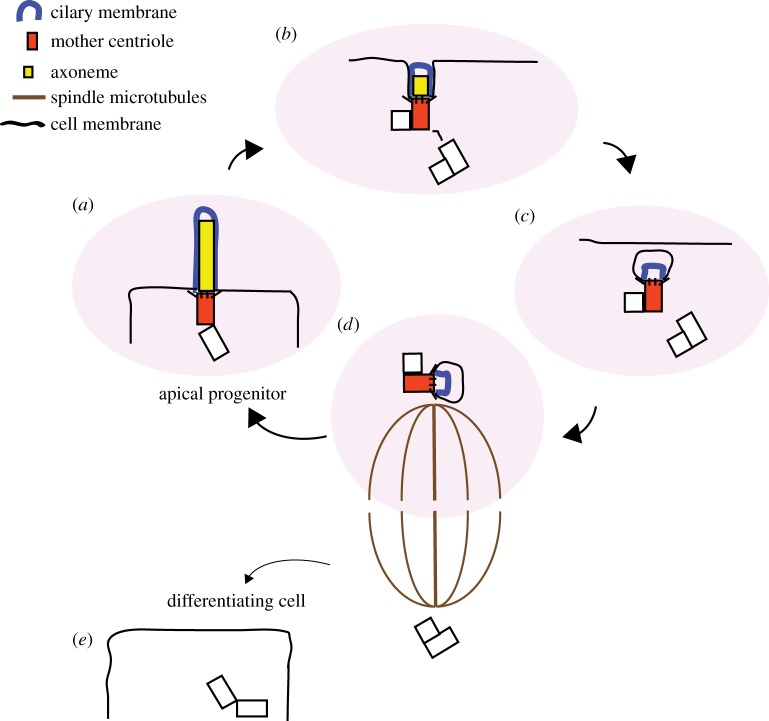
A model for mother centriole retention and stemness in mice APs. (*a*) The mother centriole (red) serves as a basal body for the cilium that is essential for cell signalling transduction. (*b*) After centriole duplication, the cilium starts to be disassembled in preparation for mitosis. (*c*) The centrosome containing the old mother centrosome is finally internalized, bringing with it a remnant of the ciliary membrane (blue). (*d*) Mitosis is asymmetric because only one daughter cell inherits the mother centrosome, which carries the ciliary membrane remnant. (*e*) The cell that lacks the ciliary membrane remnant takes longer to organize the cilium than its sister and enters the differentiation programme.

In mouse RGPs/APs, recent progress on this front links mother centriole to stemness through the retention of part of the ciliary membrane that is brought into the cell by the endocytosed mother centriole at mitosis onset and is thus delivered to one of the two daughter cells [57] (figure 3). Incidentally, the presence of the ciliary membrane remnant through mitosis challenges the long established view of the total disassembly of cilia during cell division. The presence of this ciliary membrane remnant speeds up primary cilium assembly and with it primary-cilium-dependent signal transduction that contributes to stemness. This is an excellent example of how evolution can exploit the general principle of centriolar asymmetry that is inherent to every animal cell to differentially fine tune the ability of two sister cells to respond to environmental signals in order to fulfil their roles in neural development [57]. These observations are very much along the lines proposed in 2009 by Anderson and Stearns who showed that in stable cell lines that divide ‘symmetrically’, the daughter cell receiving the older mother centriole usually grows a primary cilium and is capable of responding to Sonic hedgehog (Shh) before its sister [63]. The same asymmetry in cilium formation and Shh response was later observed in *ex vivo* cultures of mouse neuroepithelial cells [64]. The recent identification of the dismantling of the primary cilium as a key event in neuronal differentiation underscores the role of cilia in maintaining proliferation potential during neurogenesis [65]. Another interesting finding recently reported regards the role of Wingless-related integration site (Wnt) signalling in centrosome fate. In mouse embryonic SCs in culture, a localized source of Wnt3a is sufficient to drive the segregation of the mother centrosome towards the Wnt-source proximal daughter cell that expresses high levels of nuclear β-catenin and pluripotency genes [66].

Progress has also been made regarding the molecular details of centriole asymmetry control in SRAD cycles in *Drosophila* larval NBs. Five essential players have been identified: POLO, CNN, pericentrin-like protein (PLP), centrobin (CNB) and partner of inscuteable (PINS). POLO is a multipurpose Ser/Thr kinase with numerous substrates [17,30,37,67,68]. CNN is a large coiled-coil reach protein that accounts for a significant fraction of the PCM mass and that plays a fundamental role in the recruitment of γTUB complexes [18,36,69,70]. PLP, the fly homologue of human pericentrin, is also a large coiled-coil reach protein that localizes to both PCM and centrioles [36,71–74]. *Drosophila* CNB, like its human homologue (CNTROB) is a distinct daughter centriole marker [37], and co-immuno precipitates with POLO, CNN and other centriolar proteins [30]. PINS (partner of inscuteable), homologue to mPins/LGN in mammals, is a modular protein with several tetratricopeptide repeat and GoLoco domains that plays a critical role in cell cortical polarity and spindle orientation [2,3,5,11]. POLO, CNN and PLP are critical for mitotic centrosome maturation, but CNB and PINS are not.

**Figure 4.**
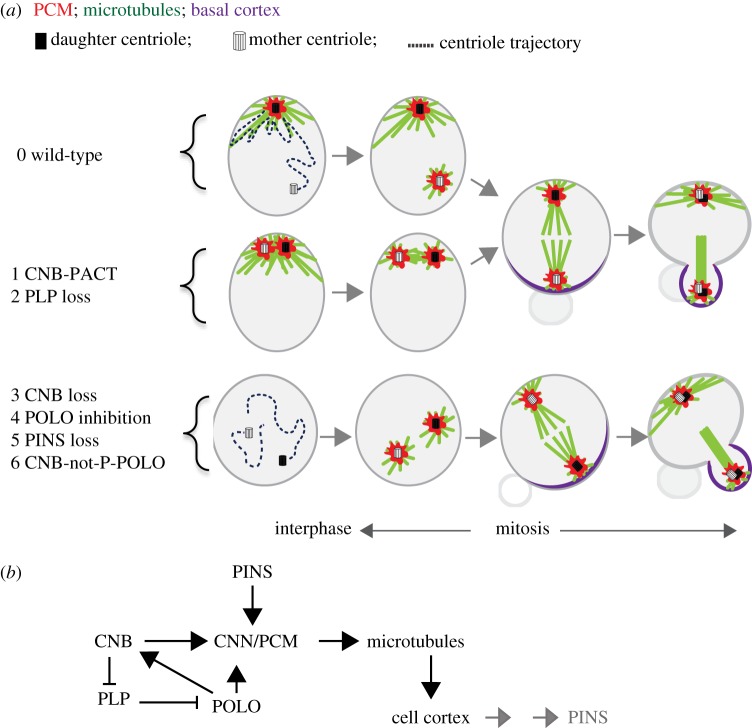
Molecular circuitry involved in centrosome asymmetry in *Drosophila* NB. (*a*) Cartoon of the phenotypic consequences of different experimental manipulations on mother–daughter centrosome behaviour. Conditions 1 and 2 equalize both centrosomes as daughters that remain apical-cortex bound and MTOC competent during interphase. Neither cortical polarization nor polarity orientation appears to be affected by these conditions. Conditions 3–6 equalize both centrosomes as mothers that remain motile and MTOC incompetent during interphase. These conditions do not affect cortical polarization but impair polarity orientation. ‘POLO inhibition’ refers to treatment with BI-2536. ‘CNB-not-P-POLO’ refers to a mutant form of CNB that cannot be phosphorylated by POLO. (*b*) Known molecular interactions. The grey arrows indicate that the cortex that interacts with the microtubules originated from the daughter centrosome during interphase is the place of assembly of different protein complexes, including PINS, during mitosis.

Experiments showing that CNB depletion in *Drosophila* NBs impedes retention of the relatively large quantities of PCM that characterize daughter centrioles during interphase, while CNB ectopic localization enables mother centrioles to do so, demonstrating that centriolar CNB is both necessary and sufficient to trigger this process [30] (figure 4). POLO's function in interphase PCM retention is at least twofold: to phosphorylate CNB, which is essential for CNN recruitment [30,37] and to phosphorylate CNN, which is essential for the recruitment of other PCM components, including of γ-tubulin (γTUB) complexes [75]. PLP is more abundant in mother than in the daughter centrioles [36,72] and comes into the equation as an inhibitor of PCM retention whose centriolar localization is negatively regulated by CNB. Consequently, PLP depletion phenocopies CNB ectopic localization enabling the mother centriole to retain PCM (figure 4) and pancentriolar CNB-PACT displaces PLP in the mother centriole that can then bind POLO and stabilize PCM [72]. The role of the fifth component, PINS, essential as it is, remains a mystery. In PINS, loss of function conditions the post-mitotic centrosome migrates to the apical cortex and soon after splits in two, just as it does in wild-type NBs. However, soon after centrosome splitting, PINS requirement becomes apparent as the daughter centrosome starts to behave like the mother, losing PCM and apical attachment [18]. Intriguingly, this phenotype can be observed at a time, early in interphase, when no sign of the PINS cortical crescent that characterizes mitotic NBs is still visible [18,30]. Furthermore, daughter centrioles detached from the cortex by microtubule poisons efficiently retain PCM. The involvement of POLO, CNN and PLP suggests that at least part of the pathway of centrosome maturation, which recruits PCM on mitotic centrosomes in most animal cells, has been co-opted to maintain mitotic-like levels of PCM on one of the centrioles throughout interphase in *Drosophila* NBs. Mitotic centrosome maturation, however, requires neither PINS, nor CNB, and is less sensitive to inhibition by the POLO inhibitor BI2536 [30] (figure 4*a*).

These results place centriolar CNB as a key factor that inhibits PLP centriolar binding, thus allowing the centriolar localization of POLO, which in turn leads to CNB phosphorylation, γTUB complex recruitment and microtubule organizing centre (MTOC) activity (figure 4*b*). It is important to stress that although most of these proteins, including CNB, are found in all sorts of cell lineages, the pathway described here has only been shown to operate in NBs. Thus, for instance, expression of the pancentriolar CNB fusion that forces the mother-to-daughter centriole behaviour in NBs does not appear to do so neither in GMCs nor in cells from wing imaginal discs [30].

## 5. A few answers among many questions

The following questions and, when available, accompanying answers summarize the current state of knowledge and future research on the topic of *correlated centriole age and fate in SRAD*.

Is it a universal principle, or is it species or just cell-type specific?

Currently available data are still fully consistent with the hypothesis of a tight link between centrosome age and fate in cells that divide through SRAD; such is the case in all five cases of SRAD in which mother–daughter centrosome fate has been determined. Indeed, based as it is in only five cell types, there is no way of predicting the likelihood of this hypothesis being a general principle, or whether it will be falsified in the next type of SRAD studied in detail. Either way, however, the case would remain open as to its functional relevance in the cell types to which it applies.

Does it always go such that it is the mother centrosome the one retained by the daughter cell with renewed stemness?

Data currently on hand demonstrate that the answer is no. In *Drosophila*, it does not even hold true for adult SCs since mGSCs retain the mother centrosome while fGSCs retain the daughter centrosome.

*Does the negative answer to the previous question imply that correlated centriole age and fate cannot contribute to non-random old-versus-new DNA strand segregation (immortal strand hypothesis*)*?*

Certainly not; of course, it could. In fact, centrosomes, through mother–daughter centrosome differences, remain the primary suspects to understand biased chromatid segregation [41]. Indeed, a recent report shows that in *Drosophila* mGSCs, X and Y sister chromatid segregation is highly biased during SRAD and that this process requires the PCM protein CNN as well as components of the linker of nucleoskeleton and cytoskeleton complex [76].

Why should the mother–daughter fate be inverse in mGSCs compared with fGSCs and NBs?

No answer has been provided so far. The recent demonstration that fGSCs, like NBs, retain the daughter centrosome complicates the issue further because it rules out a simple interpretation based on differences between adult SCs that perdure during the lifespan of the organism and cells like NBs that proliferate only at a certain period of development. To complicate the issue even further, it must be noted that vertebrate RGPs/APs that are in this regard much more related to NBs than to GSCs retain the mother centrosome.

Does correlated centriole age and fate operate in ACD that is not SRAD?

We do not have an answer to this question yet.

Altogether, the above considerations underscore the need to characterize centrosome behaviour in SRAD in as many cell types and species as possible.

Is correlated centriole age and fate necessary for SRAD and development?

The answer is clearly yes in RGPs/APs in rodents where mother centriole retention contributes to stemness [52,57].

The situation is not so clear in flies. Equalizing the two centrioles of an NB as either mothers or daughters does not appear to affect NB cortical polarity, asymmetric cortex segregation or daughter cell size difference [30,72], nor does it result in gross anatomic abnormalities in larval brains. In NBs with centrioles equalized as mothers, the site of budding of the small differentiating daughter does not coincide with the previous bud with the same precision that it does in wild-type NBs [30]. However, this phenotype might be attributable to the lack of an interphase aster rather than to the loss of centriole asymmetry because it is not observed when centrioles are equalized as daughters [30]. Indeed, cortical centrosome anchoring during interphase is essential to defining the site of apical cortical polarity, and as a consequence, to defining SRAD orientation and the site of budding of the small daughter cell [77]. SRAD does not seem to be affected by swapping centrosomes' fate either (i.e. mother centrosome inherited by the NB and daughter centrosome inherited by differentiating daughter) at least within a couple of consecutive cell cycles [77].

Could it be an epiphenomenon owing to interphase aster assembly?

*Drosophila* mGSCs and NBs retain the centrosome that during interphase remains bound to the stem-proximal side of the cortex, near the hub or the apical cortex, respectively. Such a centrosome happens to be the mother in mGSCs and the daughter in NBs. Because cortical binding is microtubule-dependent, the MTOC-capable centrosome is retained. The loss of centrosome-age-dependent fate in NBs with equalized centrioles is fully consistent with this interpretation [30]. But of course this still leaves open the crucial question of why are centrosomes made so markedly different in the first place in these cells, a question for which no epiphenomenal answer springs to mind.

## 6. Final remarks on function

If complex processes like SRAD depended critically on single pathways, most mutant conditions for genes involved in these processes would result in total disaster. However, one of the lessons derived from two decades of research on SRAD in *Drosophila* NBs in particular, as well as from many decades of research on *Drosophila* development and cell biology in general, is that complex processes are often made robust through different layers of control that are partially redundant; hence partially dispensable. The last minute correction of cortical defects and spindle alignment in a number of mutants that display prominent cortical polarity defects up to metaphase in *Drosophila* NBs, known as ‘telophase rescue’ [78], speaks well to this end and so does the recently identified basal-cortex-dependent, spindle-independent mechanism of cleavage furrow positioning [79].

While neither the elaborated process of PCM loss from the mother and stabilization over the daughter centriole nor the resulting interphase aster that is conspicuously displayed by NBs and forecasts in interphase the position of the oncoming mitotic apical cortex demonstrate function, it may be unwise to dismiss these complex processes as non-functional on the basis of soft evidence; the devil tends to be in the details [80], and most of the details on this issue we are still missing. Despite the long list of questions still standing, a phenomenon like correlated centrosome age and cell fate asymmetry is a strong advocate on behalf of centrosomes as organelles with functions in somatic cells, and, thus, a significant contributor to these days' ‘centrosomes renaissance’. After all, processes that are observed across species and are dependent on complex molecular interactions, but serve no purpose are hard to reconcile with the, this time real, Dobzhansky's principle: ‘nothing in biology makes sense except in the light of evolution’ [1].

## Funding statement

Work in our laboratory is supported by BFU2012–32522 from the Spanish MIMECO, SGR Agaur 2009 CG041413 from Generalitat de Catalunya and AdG 2011 294603 advanced grant from the European Research Council.
